# Multi‐institutional questionnaire on treatment strategies for superficial entire circumferential esophageal squamous cell carcinoma

**DOI:** 10.1002/deo2.206

**Published:** 2023-01-17

**Authors:** Tomohiro Kadota, Ryu Ishihara, Waku Hatta, Masao Yoshida, Hiromitsu Kanzaki, Daisuke Kikuchi, Yoichiro Ono, Seiichiro Abe, Yoshinobu Yamamoto, Toshiyuki Yoshio, Yuji Urabe, Naoyuki Yamaguchi, Yasuaki Nagami, Toshiro Iizuka, Hiroaki Takahashi, Tsuneo Oyama, Tomonori Yano

**Affiliations:** ^1^ Department of Gastroenterology and Endoscopy National Cancer Center Hospital East Chiba Japan; ^2^ Department of Gastrointestinal Oncology Osaka International Cancer Institute Osaka Japan; ^3^ Division of Gastroenterology Tohoku University Graduate School of Medicine Miyagi Japan; ^4^ Division of Endoscopy Shizuoka Cancer Center Shizuoka Japan; ^5^ Department of Gastroenterology and Hepatology Okayama University Graduate School of Medicine, Dentistry and Pharmaceutical Sciences Okayama Japan; ^6^ Department of Gastroenterology Toranomon Hospital Tokyo Japan; ^7^ Department of Gastroenterology Fukuoka University Chikushi Hospital Fukuoka Japan; ^8^ Endoscopy Division National Cancer Center Hospital Tokyo Japan; ^9^ Department of Gastrointestinal Oncology Hyogo Cancer Center Hyogo Japan; ^10^ Department of Gastroenterology Cancer Institute Hospital Tokyo Japan; ^11^ Division of Regeneration and Medicine Center for Translational and Clinical Research Hiroshima University Hospital Hiroshima Japan; ^12^ Department of Gastroenterology and Hepatology Nagasaki University Hospital Nagasaki Japan; ^13^ Department of Gastroenterology Osaka City University Graduate School of Medicine Osaka Japan; ^14^ Department of Gastroenterology Tokyo Metropolitan Cancer and infectious Diseases Center Komagome Hospital Tokyo Japan; ^15^ Department of Gastroenterology Keiyukai Daini Hospital Hokkaido Japan; ^16^ Department of Endoscopy Saku Central Hospital Advanced Care Center Nagano Japan

**Keywords:** chemoradiotherapy, entire circumferential esophageal squamous cell carcinoma, endoscopic submucosal dissection, esophagectomy, local steroid injection

## Abstract

**Objectives:**

Recent innovations in prophylactic treatment with steroids have overcome the issue of esophageal stricture after endoscopic submucosal dissection (ESD), except in entire circumferential esophageal squamous cell carcinoma (EC‐ESCC). Current Japanese guidelines weakly recommend performing ESD for clinical epithelial/lamina propria EC‐ESCC with a longitudinal extension <50 mm upon implementing prophylactic treatment against stricture. However, the accurate indications for ESD in EC‐ESCC remain unknown, and strategies differ among institutions. The aim of this study was to understand the initial treatment strategy for EC‐ESCC and prophylactic treatment after ESD against esophageal stricture.

**Methods:**

A questionnaire survey was conducted across 16 Japanese high‐volume centers on the initial treatment for EC‐ESCC according to the invasion depth and longitudinal extension, and prophylactic treatment against stricture.

**Results:**

ESD was performed as the initial treatment not only in clinical epithelial/lamina propria lesions <50 mm (88–94% of institutions), but also in clinical epithelial/lamina propria ≥50 mm (44–50% of institutions) and clinical muscularis mucosae/SM1 (submucosal invasion depth invasion within 200 μm) lesions <50 mm (56–75% of institutions). Regarding prophylactic treatment against esophageal stricture, although there was a common point of local steroid injection, the details and administration of other treatments varied among institutions.

**Conclusions:**

As ESD was performed with expanded indications for EC‐ESCC than those recommended by the guidelines in more than half of the institutions, the validity of ESD for expanded EC‐ESCC needs to be clarified. For that, it is necessary to prospectively collect short‐ and long‐term outcomes after ESD and other treatments, including esophagectomy or chemoradiotherapy.

## INTRODUCTION

In recent times, a large number of early esophageal squamous cell carcinomas (ESCCs) are treated with endoscopic submucosal dissection (ESD) worldwide. Esophageal stricture is known to be a crucial problem, especially in cases with post‐ESD mucosal defects larger than three‐quarters of the circumference of the esophageal lumen, when no prophylactic treatment to prevent stricture is administered.^1^, ^2^, ^3^ Therefore, esophageal strictures can be overcome via innovative prophylactic treatments with steroids, except in cases of entire circumferential ESCC (EC‐ESCC).^4^, ^5^, ^6^ In cases of EC‐ESCC, some previous reports have demonstrated that the stricture rate remains high (62%–100%), even with local steroid and/or oral steroid administration.^7^, ^8^, ^9^, ^10^, ^11^ In particular, pre‐ESD longitudinal extension longer than 50 mm has been reported to be a risk factor for refractory stricture.^9^, ^10^ Consequently, there is only a weak recommendation for endoscopic resection in patients with clinical (c) T1a‐epithelial (EP)/lamina propria (LPM) EC‐ESCC with a longitudinal extension of less than 50 mm, upon implementation of prophylactic treatment against stricture in the ESD/endoscopic mucosal resection (EMR) guidelines for esophageal cancer.^12^ Moreover, the proportion of non‐curative resection after ESD for superficial EC‐ESCC is reported to be relatively high (17%–39%) in EC‐ESCC.^8^, ^9^, ^10^ In addition, a report focusing on the cases with cMM/SM1 EC‐ESCC resected by ESD or surgery reported that as many as 86% (12/14) of cases showed pathological results corresponding to non‐curative resection.^13^ Thus, endoscopic resection is not recommended for cT1a‐MM/cT1b‐SM1 (submucosal invasion depth invasion within 200 μm) EC‐ESCC, considering the possibility of non‐curative resection after ESD and the influence of esophageal stricture after ESD on additional treatment.^12^

Esophagectomy or definitive chemoradiotherapy (CRT) is generally recommended for cT1 ESCC if ESD is not performed.^14^ Although one prospective study (JCOG0502) has reported on a comparison between esophagectomy and definitive CRT for cT1b‐SM ESCC, regardless of the circumference of the lesion, there are no reports focusing on cT1 EC‐ESCC.

There are no reports on the initial treatment selection for superficial EC‐ESCC according to the lesion's invasion depth and longitudinal extension in real clinical practice. Also, little is known about the current status of the prophylactic treatment against stricture after ESD of EC‐ESCC although there were some reports from high‐volume centers.^6^, ^11^, ^15^, ^16^, ^17^ It seemed to be necessary to understand these current situations when considering the expansion of ESD indication for EC‐ESCC in the future. Thus, the aim of this study was to understand the treatment strategy of Japanese high‐volume centers that treat a relatively large number of EC‐ESCC, in terms of initial treatment for superficial EC‐ESCC and prophylactic treatment against esophageal stricture after ESD.

## METHODS

**FIGURE 1:**
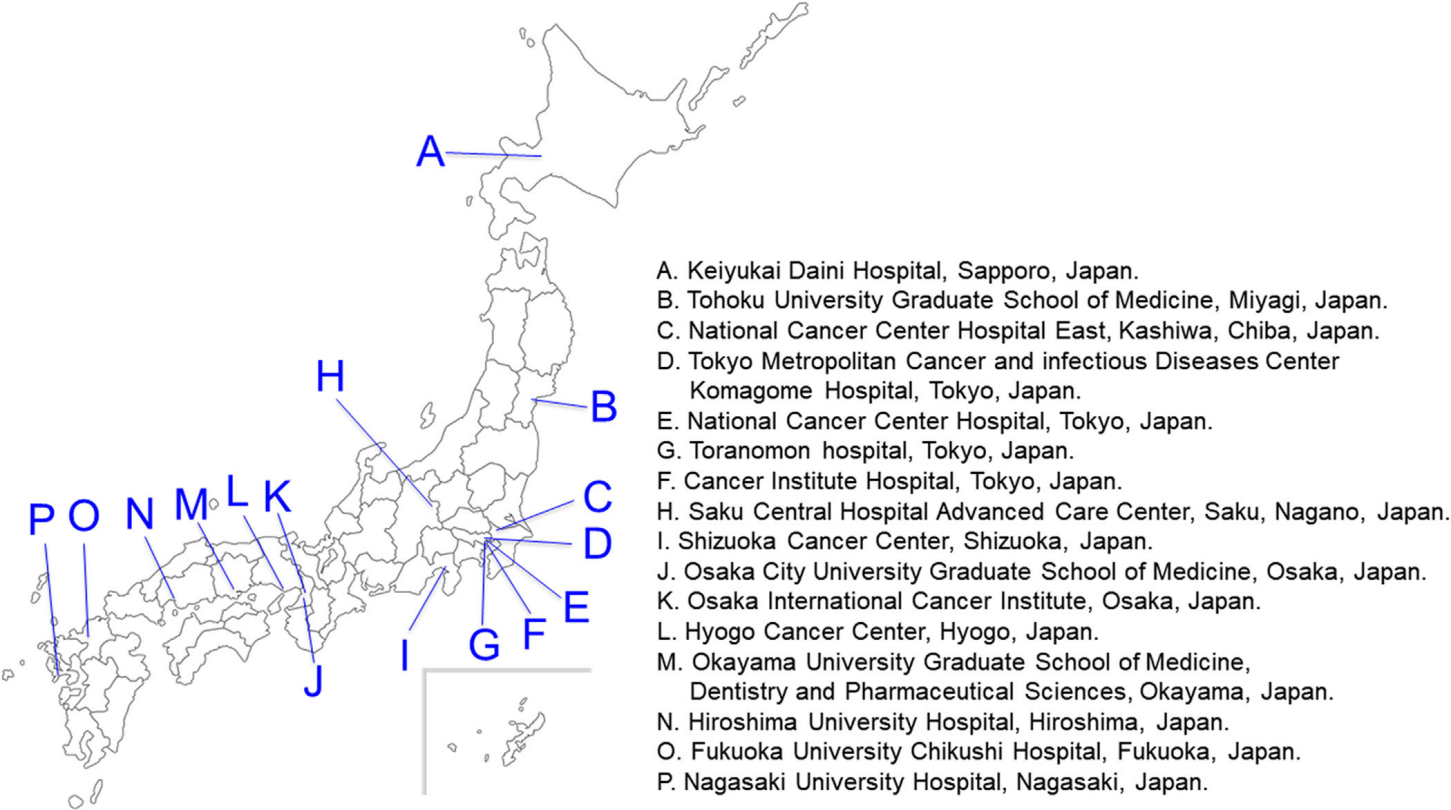
The participating institution

This was a questionnaire survey of 16 Japanese high‐volume centers all over Japan, which are recognized as the training facilities for certified fellows of endoscopists by the Japan Gastroenterological Endoscopy Society and the major referral institutions treating a large number of esophageal cancers (most centers performing with more than 50 cases of esophageal ESD per year; Figure 1). On May 14, 2020, the questionnaire was sent via email to a representative doctor in each institution, requesting the institutional treatment strategy and retrieved from all institutions by June 28, 2020. In addition, informed consent was obtained from participating doctors for the publication of this survey results.

As shown in Table 1, information regarding the initial treatment for superficial EC‐ESCC according to preoperative invasion depth, longitudinal extension, and patient condition (Q1 and Q2) was requested. Information regarding prophylactic treatment against esophageal stricture (Q3) and treatment for esophageal stricture was also requested.

**TABLE 1:** Contents of the questionnaire

Q1: What is the initial treatment in your institution for the following types of superficial entire circumferential esophageal squamous cell carcinoma (EC‐ESCC) (12 lesions*) in patients aged <75 years with a physical condition suitable for surgery?
Q2: What is the initial treatment in your institution for the following types of superficial EC‐ESCC (12 lesions*) in patients aged >75 years or with a physical condition not suitable for surgery?
*12 lesions had the preoperative depth of cEP/LPM, cMM/DM1, or cSM2 and the longitudinal extension of the lesion of < 30 mm, 30 mm <, < 50 mm, or 50 mm <.
Please answer from the following (i) – (iii) options.
Endoscopic submucosal dissection (ESD) for almost all lesions. ESD in case of refusal to surgery and chemoradiotherapy (CRT). Surgery or CRT.
Q3: Which prophylactic treatment against esophageal stricture is performed in your institution for the following types of mucosal defect after esophageal ESD (4 lesions**) in patients who can take steroid medication?
**12 lesions had the longitudinal extension of the post‐ESD mucosal defect of < 30 mm, 30 mm <, < 50 mm, 50 mm <, < 70 mm or 70 mm <.
Please answer according to the prophylactic treatment including local steroid injection, oral steroid administration, prophylactic endoscopic balloon dilation, and others.
Q3‐1: Local steroid injection
Q3‐1‐1: Is a local steroid injection administered? (i) yes, (ii) no.
Q3‐1‐2: If yes, how often are local steroid injections administered?
Only once (ii) More than twice premeditatedly sometimes more than twice according to situation (iv).
Q3‐1‐3: What is the dose of the injected steroid (triamcinolone acetonide) per session?
(i) 40–100 mg (ii) 101–200 mg (iii) 201–300 mg (iv) 301 mg or more
Q3‐2: Oral steroid administration
Q3‐2‐1: Are oral steroids administered? (i) yes, (ii) no.
Q3‐2‐2: If yes, what is the dose and schedule of oral steroid (prednisolone) administration?
started at 30 mg/day and tapered gradually over 8 weeks started at 30 mg/day and tapered gradually over 12 weeks. other
Q3‐3: Prophylactic endoscopic balloon dilation (EBD), which is defined as dilation up to 15 or 18 mm when an ordinary‐sized endoscope can pass through the post‐ESD site.
Q3‐3‐1: Is prophylactic endoscopic balloon dilation performed? (i) yes, (ii) no.
Q3‐3‐2: If yes, please provide the details of prophylactic endoscopic balloon dilation, such as timing.
Q3‐4: Other
Q3‐4‐1: Are other prophylactic treatments performed? (i) yes, (ii) no.
Q3‐4‐2: If yes, please provide the details of other prophylactic treatments.
Q4: Which treatments are performed if esophageal stenosis occurs?
Please answer according to treatments for stricture including EBD, endoscopic radial incision and cutting (RIC), stent placement, etc.
Q4‐1: Endoscopic balloon dilation, which is defined as dilation when an ordinary‐sized endoscope cannot pass through the post‐ESD site.
Q4‐1‐1: Is endoscopic balloon dilation performed? (i) yes, (ii) no.
Q4‐1‐2: If yes, how many weeks is endoscopic balloon dilation performed for?
Q4‐1‐3: Is local steroid injection immediately after endoscopic balloon dilation performed?
(i) yes (ii) no
Q4‐1‐4: If yes, what is the dose of the injected steroid (triamcinolone acetonide) per session?
Q4‐2: Endoscopic RIC
Q4‐2‐1: Is endoscopic RIC performed? (i) yes, (ii) no.
Q4‐2‐2: If yes, what is the reason for performing endoscopic RIC?
Q4‐3: Stent placement
Q4‐3‐1: Is stent placement performed? (i) yes, (ii) no.
Q4‐3‐2: If yes, what kind of stents are used?
Q4‐3‐3: What about the placing of stents?
Q4‐4: Other
Q4‐4‐1: Are other treatments for stricture performed? (i) yes, (ii) no.
Q4‐4‐2: If yes, please provide the details of the other treatments.

## RESULTS

We received responses from all 16 institutions, and there were no institutions that did not respond. The respondents consisted of 16 expert endoscopists from 16 Japanese high‐volume centers, including six university hospitals, six cancer centers, and four general hospitals.

### Initial treatment strategy for superficial EC‐ESCC

**FIGURE 2:**
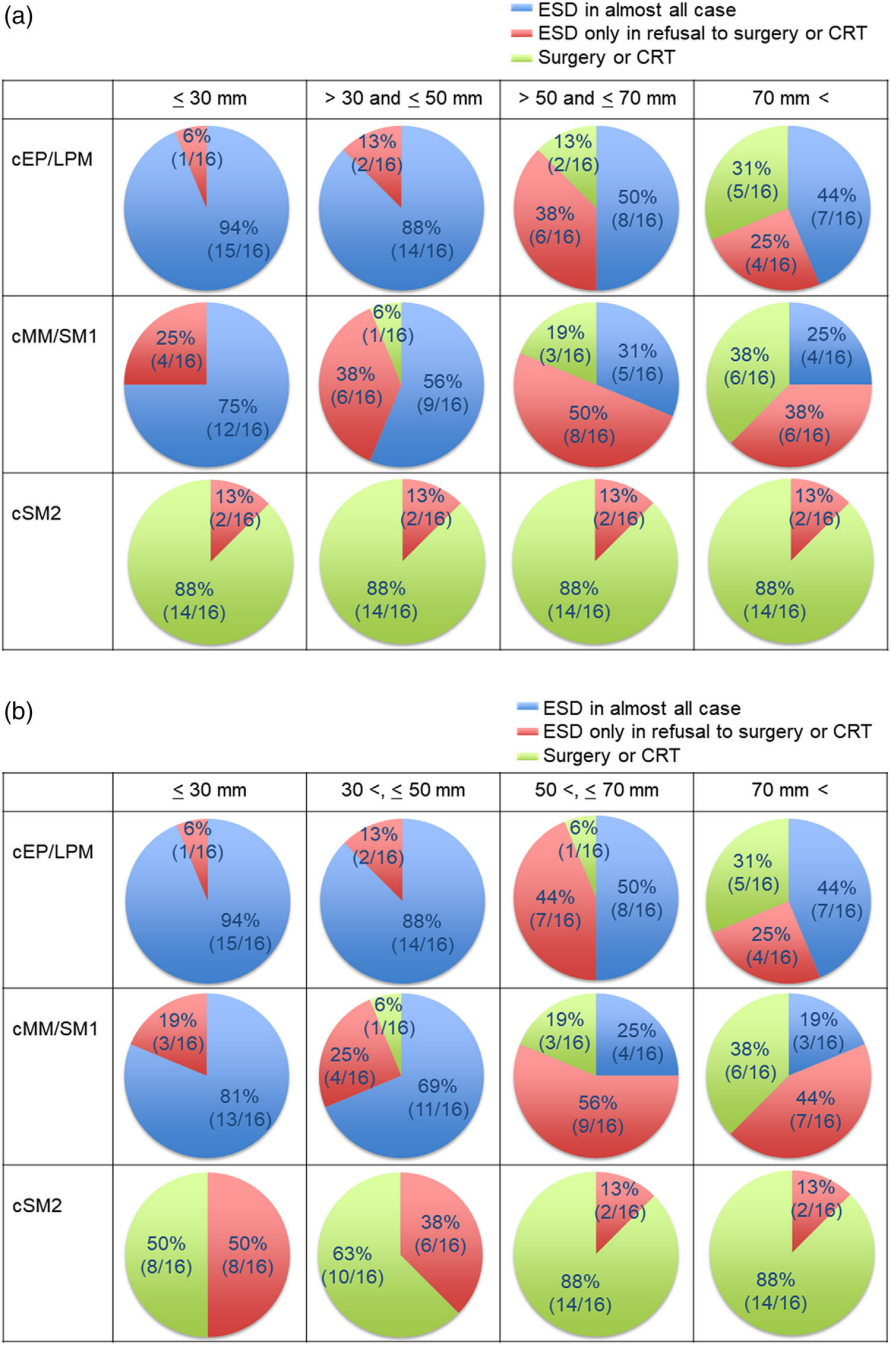
(a) Initial treatment in case of patients under 75 years old with a physical condition suitable for surgery according to preoperative invasion depth and longitudinal extension. (b) Initial treatment in case of patients over 75 years old or with a physical condition not suitable for surgery according to preoperative invasion depth and longitudinal extension

Information about the initial treatment strategy for superficial EC‐ESCC was requested based on the invasion depth and longitudinal extension of the lesion and the patient's background (Table 1, Q1 and Q2). As shown in Figure 2a, in patients aged less than 75 years with a physical condition suitable for surgery, cEP/LPM lesions with a longitudinal extension of 50 mm or less and cMM/SM1 lesions with a longitudinal extension of 30 mm or less were mainly treated with ESD at most institutions (88–94% and 75%). cEP/LPM lesions with a longitudinal extension of more than 50 mm and cMM/SM1 lesions with a longitudinal extension of more than 30 mm and 50 mm or less were mainly treated with ESD at half of the institutions (44%–50% and 56%). cMM/SM1 lesions with a longitudinal extension of more than 50 mm were mainly treated with ESD at some institutions (25%–31%). cSM2 lesions were seldom treated with ESD, regardless of the lesion's longitudinal extension in most institutions. These results were similar to those in patients older than 75 years or with physical conditions not suitable for surgery (Figure 2b).

### Prophylactic treatment strategy

**FIGURE 3:**
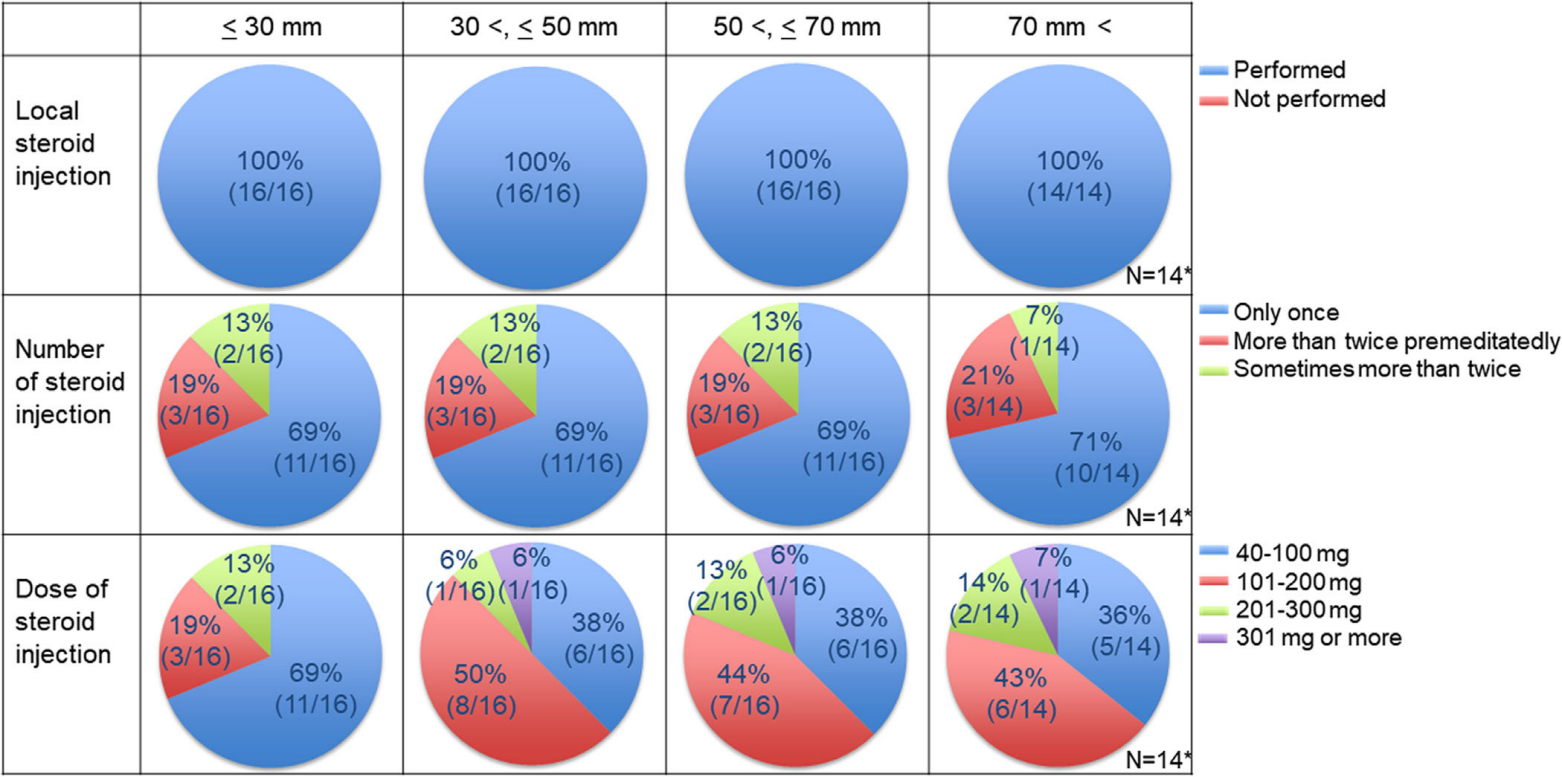
Local steroid injection according to the longitudinal extension of the lesion. *: Two institutions did not respond to lesions with a longitudinal extension of more than 70 mm because they did not treat with endoscopic submucosal dissection (ESD) at all.

The prophylactic treatment strategy against esophageal stricture after ESD was requested according to the lesion's longitudinal extension (Table 1, Q3). As shown in Figure 3, local steroid injections were routinely performed regardless of the longitudinal extension of the lesion in all institutions. The number of local steroid injections did not differ based on the longitudinal extension of the lesion, and a single injection session was performed in most institutions (69%–71%). The amount of locally injected steroid differed based on the longitudinal extension of the lesion. Seventy‐two percent of institutions used 40–100 mg of triamcinolone acetonide per session for lesions with a longitudinal extension of 30 mm or less. In contrast, 40–100 mg (36%–38% of institutions) and 101–200 mg (43%–50% of institutions) of triamcinolone acetonide were used for lesions with a longitudinal extension of more than 30 mm.

**FIGURE 4:**
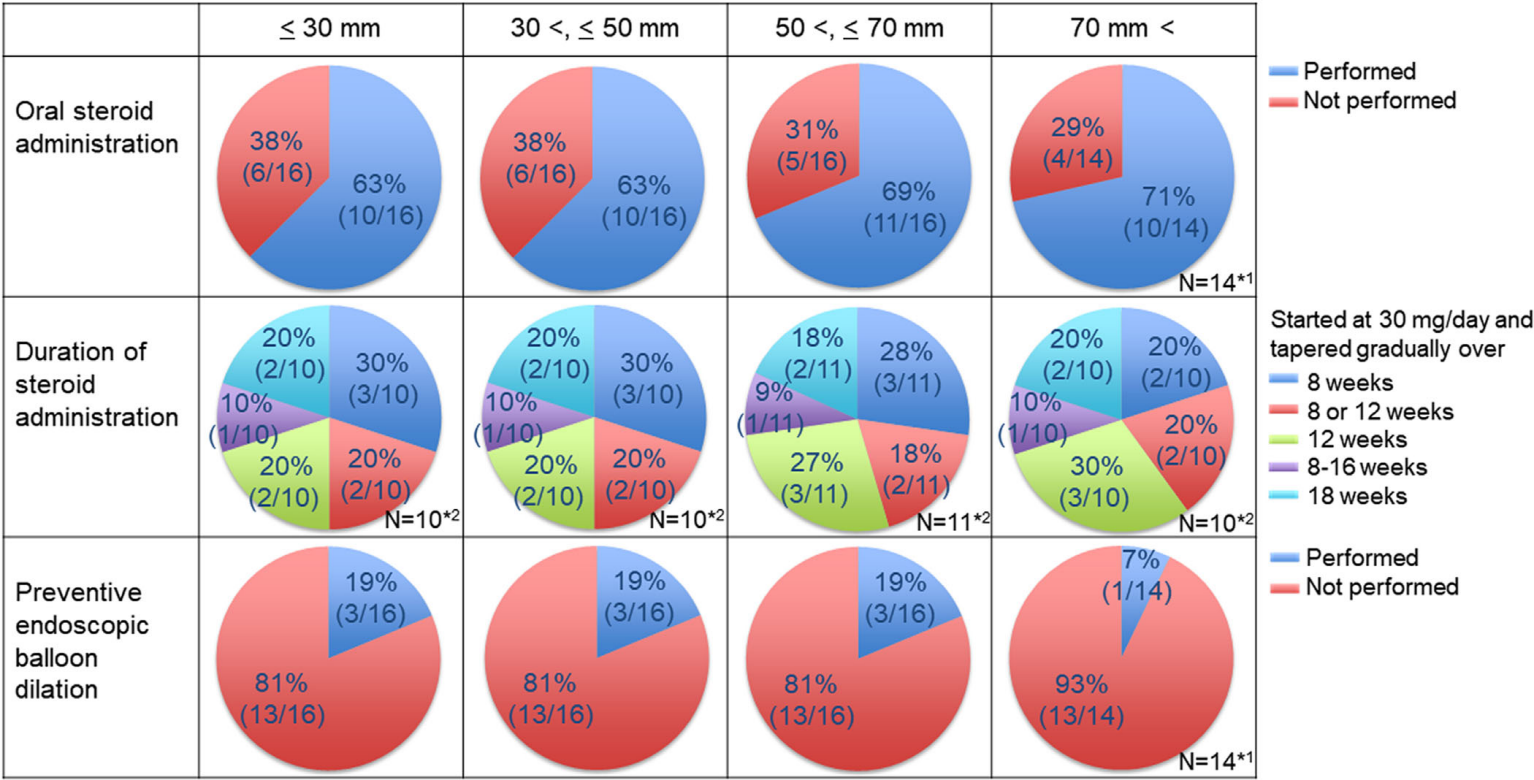
Oral steroid administration and prophylactic endoscopic balloon dilation according to the longitudinal extension of the lesion. *1: Two institutions did not respond to lesions with a longitudinal extension of more than 70 mm because they did not treat with endoscopic submucosal dissection (ESD) at all. *2: Question as to the duration of steroid administration was responded to by institutions using oral steroid administrations (10 or 11 institutions).

Regarding oral steroid administration, many institutions (63%–71%) used oral steroids, regardless of the longitudinal extension of the lesion (Figure 4). The various regimens, centered on the regimen that was initiated at a dose of 30 mg/day and tapered for 8 weeks, were selected based on the lesions and the physician's choice.

Prophylactic endoscopic balloon dilation (EBD) was used in a few institutions (7%–19%), and this did not depend on the longitudinal extension of the lesion. In addition, one institution used polyglycolic acid sheets as a prophylactic treatment.

### Treatment strategy for esophageal stricture

**FIGURE 5:**
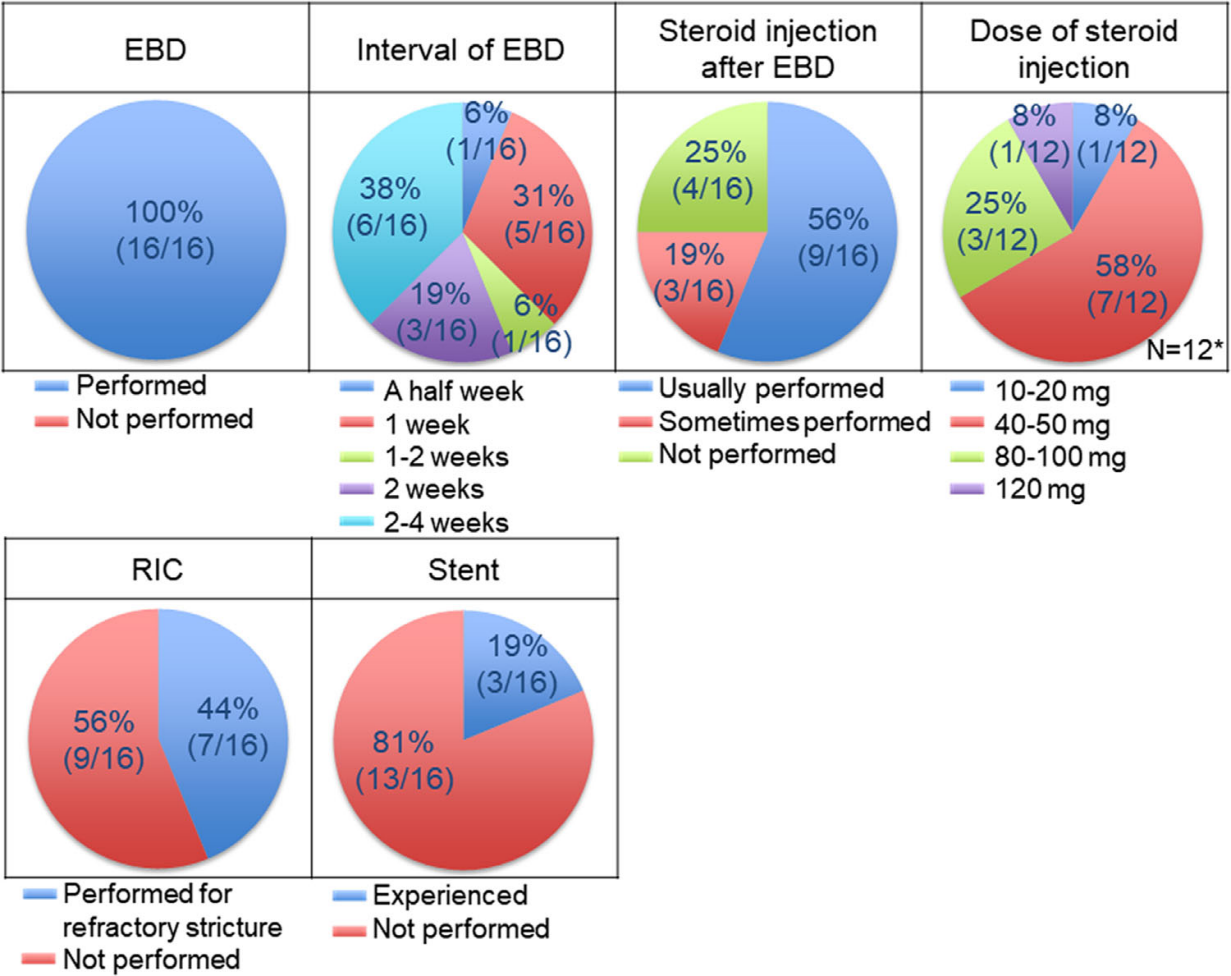
Treatment for esophageal stricture after endoscopic submucosal dissection (ESD). *: Question as to the duration of steroid injection was responded to by institutions using steroid injection after endoscopic balloon dilation (EBD; 12 institutions).

The treatment strategy for esophageal stricture after ESD was requested (Table 1 and Q4). As shown in Figure 5, EBD was performed for esophageal stricture after ESD in all institutions, and their intervals were mainly 2 weeks. Local steroid injection immediately after EBD was routinely performed in about half of the institutions (56%). The dose of steroids was often 40–50 mg (58%). Endoscopic radial incision and cutting (RIC) was performed in about half of the institutions (44%), mainly in cases of EBD‐refractory stricture. Stent placement was not routinely performed in all institutions. Although not routinely used, several institutions used self‐expandable metallic stents or biodegradable stents for the treatment of esophageal strictures after ESD.

## DISCUSSION

In this multi‐institutional questionnaire study, we demonstrated that ESD was performed with expanded indications for EC‐ESCC than those recommended by the ESD/EMR guidelines in more than half of the institutions surveyed. Regarding the prophylactic treatment against esophageal stricture, although there was a common point of local steroid injection, the details of local steroid injection and other treatments varied between institutions. As for the treatment of esophageal stricture after ESD, although EBD was the main treatment, RIC was also performed in half of the institutions for refractory stricture.

The results of this questionnaire indicated that the initial treatment strategy for EC‐ESCC remains controversial. This is because of the lack of short‐ and long‐term outcomes data for each treatment. In particular, there are several diagnostic and therapeutic factors that may suggest that ESD should not be performed for lesions of the entire circumference. The invasion depth of EC‐ESCC is more difficult to diagnose than that of non‐entire circumferential ESCC, and relatively high non‐curative resection rates (17%–39%)^7^, ^8^, ^9^, ^10^ have been reported if ESD was selected, which require additional treatment. Conversely, the inaccurate depth diagnosis could also cause overtreatment if esophagectomy is selected. In addition, as a therapeutic factor, stricture after ESD occurs frequently and tends to be refractory even if prophylactic treatment is performed. Many studies have reported case series of ESD for EC‐ESCC without serious adverse events except for stricture, and the prevalence of patients treated with ESD is increasing in some institutions. Based on the risk‐benefit balance from limited retrospective studies, only cEP/LPM EC‐ESCC with a longitudinal extension of 50 mm or less was recommended weakly for performing ESD by the ESD/EMR guidelines. In fact, the guidelines provided no detailed recommendation for performing ESD of other EC‐ESCC including a longitudinal extension of more than 50 mm and cMM/SM1 EC‐ESCC. Moreover, there is an oncological issue if selecting ESD. As ESD is only a local treatment, the pathological evaluation after ESD does not completely reflect the prognosis. And, there is no relevant survival data of patients who were treated with ESD for longitudinal extension more than 50 mm and cMM/SM1 EC‐ESCC. Therefore, we believe that multicenter prospective studies are warranted to evaluate the diagnostic accuracy of invasion depth and the short‐ and long‐term oncological outcomes after ESD for EC‐ESCC, which could validate and potentially expand its indications in the guidelines. Since there were no comparative data of treatment outcomes after surgery or CRT for EC‐ESCC, it was also considered necessary to collect them prospectively.

Local steroid injection and oral steroid administration are mainly used for prophylactic treatment of esophageal stricture after ESD; however, the details have not yet been determined. Therefore, the superiority of oral steroid administration in terms of stricture‐free survival over local steroid injection therapy after ESD is currently being evaluated in patients with ESCC in a phase III randomized controlled trial (JCOG1217).^18^ However, as EC‐ESCC is not targeted in JCOG1217, the appropriate prophylactic treatment for EC‐ESCC remains unresolved. In addition, although this multi‐institutional questionnaire did not focus on the complications related to these treatments, many complications have been reported,^19^, ^20^, ^21^, ^22^ and it does not seem feasible to simply increase the dose and frequency of steroids in terms of efficacy and safety balance. Therefore, it is also important to identify the optimal prophylactic treatment after ESD for EC‐ESCC in a prospective multi‐institutional study.

Although there have been many previous reports on the treatment for esophageal stricture after esophagectomy for ESCC, there are few reports focusing on esophageal stricture after ESD. It is well known that the length of stricture after ESD is longer than that after esophagectomy, and stricture after ESD is considered to be more severe than stricture after esophagectomy, with a higher refractory stricture rate and a longer time to relief from stricture.^3^ Based on the result of a randomized controlled study that reported that the local steroid injection after EBD decreased the number of EBD and reduced the duration of dysphagia after EBD for refractory stricture after esophagectomy compared to repeated EBD, local injection after EBD is also often performed for stricture after ESD.^23^ In addition, although there have been only two case reports of several patients who underwent RIC for refractory stricture after ER,^24^, ^25^ RIC is sometimes performed for refractory stricture after ESD. It turned out that local injection after EBD for stricture after ESD and RIC for refractory stricture after ESD was performed as well as after esophagectomy in half of the institutions in this multi‐institutional questionnaire. If the efficacy of these treatments is shown, local injection after EBD and RIC may be performed as a standard treatment in the future.

This study has several limitations. First, this multi‐institutional questionnaire targeted expert endoscopists in a high‐volume center. A high volume of referral hospitals that participated in this survey have several specialists containing surgeons, advanced endoscopists, medical oncologists, and radiation oncologists and they can communicate about patients’ treatment strategies through the cancer board. Although patients with esophageal cancer tend to be treated in high‐volume centers in Japan, the generalization of our results into general hospitals can be one of the limitations of our study. Second, this multi‐institutional questionnaire did not focus on the complications of prophylactic treatments as we did not have access to patients’ clinical information. Therefore, we could not compare each prophylactic treatment. Third, there were no survey items related to surgery or CRT, and we could not compare the advantages and disadvantages of the initial treatment. Finally, as this study was a questionnaire survey, we did not assess the clinical outcomes and prognosis after ESD and after prophylactic treatment against stricture. To overcome these limitations, a multicenter prospective study is required. Hence, a prospective observational study (Outcome study for entire circumferential superficial esophageal cancer; UMIN000043763) that evaluates the clinical course after ESD, surgery, and CRT for superficial EC‐ESCC is currently being conducted by our group.

In conclusion, in this multi‐institutional questionnaire study, in more than half of the institutions, ESD was performed with expanded indications for EC‐ESCC lesions than those recommended by the ESD/EMR guidelines. In addition, the details of prophylactic treatment against esophageal strictures varied among institutions. Thus, a prospective study collecting the short‐ and long‐term outcomes after each treatment (ESD, CRT, and surgery) is needed to clarify these outcomes.

## CONFLICT OF INTEREST

Co‐authors Seiichiro Abe and Waku Hatta are associate editors of DEN Open. The rest of the authors declare no conflict of interest.
